# Impact of a Personal Health Record Intervention Upon Surveillance Among Colorectal Cancer Survivors: Feasibility Study

**DOI:** 10.2196/34851

**Published:** 2022-08-11

**Authors:** Eric Vachon, Bruce W Robb, David A Haggstrom

**Affiliations:** 1 School of Nursing Indiana University Indianapolis, IN United States; 2 Center for Health Services Research Regenstrief Institute Indianapolis, IN United States; 3 Department of Surgery School of Medicine Indiana University Indianapolis, IN United States; 4 Division of General Internal Medicine and Geriatrics School of Medicine Indiana University Indianapolis, IN United States

**Keywords:** personal health record, colorectal cancer survivors, surveillance, health record, survivor, cancer, oncology, colorectal, United States, North America, feasibility, web-based, patient belief, patient attitude, survival

## Abstract

**Background:**

There are currently an estimated 1.5 million individuals living in the United States with colorectal cancer (CRC), and although the 5-year survival rate has increased, survivors are at risk for recurrence, particularly within the first 2-3 years after treatment. National guidelines recommend continued surveillance after resection to identify recurrence early on. Adherence among survivors ranges from 23% to 94%. Novel interventions are needed to increase CRC survivors’ knowledge and confidence in managing their cancer and thus to increase adherence to follow-up surveillance.

**Objective:**

The objective of this study is to develop and test the feasibility and efficacy of a stand-alone, web-based personal health record (PHR) to increase surveillance adherence among CRC survivors, with patient beliefs about surveillance as secondary outcomes.

**Methods:**

A pre- and postintervention feasibility trial was conducted testing the efficacy of the colorectal cancer survivor (CRCS)–PHR, which had been previously developed using an iterative, user-centered design approach.

**Results:**

The average age of the sample was 58 (SD 9.9) years, with 57% (16/28) male and the majority married (20/28, 71%) and employed full-time (15/28, 54%). We observed a significant increase in adherence to colonoscopy (before: 11/21, 52% vs after: 18/21, 86%; *P*=.005) and CEA (14/21, 67% vs 20/21, 95%; *P*=.01), as well as a slight increase in CT scans (14/21, 67% vs 18/21, 86%; *P*=.10). The only significant impact on secondary outcome (patient beliefs) was benefits of CEA test (*P*=.04), as most of the beliefs were high at baseline.

**Conclusions:**

This feasibility study lays the groundwork for continued development of the CRCS-PHR to increase CRC surveillance. Patient-centered technologies, such as the CRCS-PHR, represent an important potential approach to improving the receipt of guideline-concordant care and follow-up surveillance, and not just for CRC survivors. Researchers should continue to develop patient-centered health technologies with clinician implementation in mind to increase patient self-efficacy and surveillance adherence.

## Introduction

There are an estimated 1.5 million individuals living in the United States with colorectal cancer (CRC), and an estimated 150,000 new cases will be diagnosed during 2021 [1]. While the 5-year survival rate has increased to 65%, survivors are still at risk for cancer recurrence with >40% developing recurrent disease within 5 years, and 80% of recurrences happening within the first 2-3 years after treatment. National professional guidelines recommend follow-up or surveillance tests such as colonoscopy, carcinoembryonic antigen (CEA), and computed tomography (CT) scans at specific intervals after treatment [2-4]. Survivor adherence to recommended surveillance is often poor and ranges between 23% and 94% [5-11]. Novel interventions to increase guideline-concordant surveillance, thus, are needed to improve the quality of care and outcomes among cancer survivors. In the United States, national incentives for offering access to electronic personal health records (PHRs) have promoted patient engagement through information technology [12-17]. Patient-centered health-information technologies are potentially valuable tools for survivorship care planning to increase knowledge about surveillance tests, self-efficacy, and ultimately adherence to guideline-concordant surveillance [16-21].

Web-based technologies have the capacity to reach large numbers of patients efficiently. PHR use has expanded over time [22-25], with functions developed for individuals with various chronic diseases and across the lifespan [17,26-29]. These functions may vary widely depending on the context of PHR implementation. While there exists a variety of PHRs, there are three primary categories of PHRs, which are as follows: (1) stand-alone PHRs, which do not directly connect with any other electronic systems or networks; (2) tethered PHRs, often referred to as patient portals or web-based portals, which connect with the web-based network and electronic medical record of a specific institution; and (3) integrated PHRs, which are able to connect to multiple data networks and institutions [17,19,21]. While these different types of PHR categories describe general trends, there is a fair amount of overlap in terms of functionalities. PHRs have the potential to engage patients with cancer and cancer survivors to play a more active role in their surveillance care and to increase self-efficacy and knowledge about surveillance [16-21]. Providing patients access to their own health information, management strategies, web-based resources, and communication tools with providers can increase self-management and the quality of patient-provider communication, which lead to better patient outcomes [16,17,19,21]. However, PHRs tailored to the needs of cancer survivors have not been widely developed or tested for certain cancer site populations, including CRC. Among patients with cancer, PHRs have been mainly developed that target patients with breast and lung cancer as well as breast and lung cancer survivors [30,31]. Technology-based interventions have been limited in targeting CRC survivors who are at substantial risk for recurrent disease within the first couple of years after treatment and may benefit from the use of technology-based interventions. Given these issues, there is a considerable need for the development of technology-based interventions, such as PHRs, targeted toward patients with CRC, particularly to increase adherence to the recommended surveillance [16,17,19,21].

The purpose of this pilot trial was to test the feasibility and evaluate the effectiveness of a stand-alone PHR in increasing surveillance testing (colonoscopy, CEA, and CT scan) among CRC survivors. Moreover, the study assessed the impact of the PHR upon the secondary outcomes of patient self-efficacy, knowledge regarding surveillance, and CRC patients’ perceptions of benefits and barriers to surveillance testing. The Colorectal Cancer Survivor (CRCS)–PHR was designed using open-source software to increase guideline-concordant CRC surveillance by delivering patients reminders and tracking tools regarding surveillance tests for which they were eligible. This work has the potential to benefit both researchers focused on developing technology-based interventions for patients, particularly cancer survivors, as well as clinicians who are working toward increasing adherence to guidelines.

## Methods

### Colorectal Cancer Survivors’ Personal Health Record

We developed the CRCS-PHR as a stand-alone, web-based tool for patient convenience, portability, and dissemination potential. An iterative, user-centered design approach was followed during development, including the creation of clinical content, program design, and web design usability testing. The design process began with the creation of content and technical parameters, culminating in a web-based interactive prototype. Product development included the application of established usability methods [32]. Stakeholders (consisting of patients, caregivers, and health care providers) were asked to participate in scenario-based evaluations with direct observation and debriefing interviews to gather data on user performance and preferences as described elsewhere [33]. Changes were not made in the web-based design until data from at least 4-6 stakeholders had been collected; consistent with previous work by Nielsen et al [34], a total of 17 stakeholders participated in data collection.

The CRCS-PHR includes the following clinical information: CRC surveillance guidelines, treatment received (surgery; adjuvant therapy; and lab, radiology, and procedure results), and potential future toxicities of the treatment received. The CRCS-PHR also had the ability to collect personal observations from the CRC survivor in an electronic journal or blog, relationships with providers and family members or friends, and communities with other CRC survivors.

**Figure 1:**
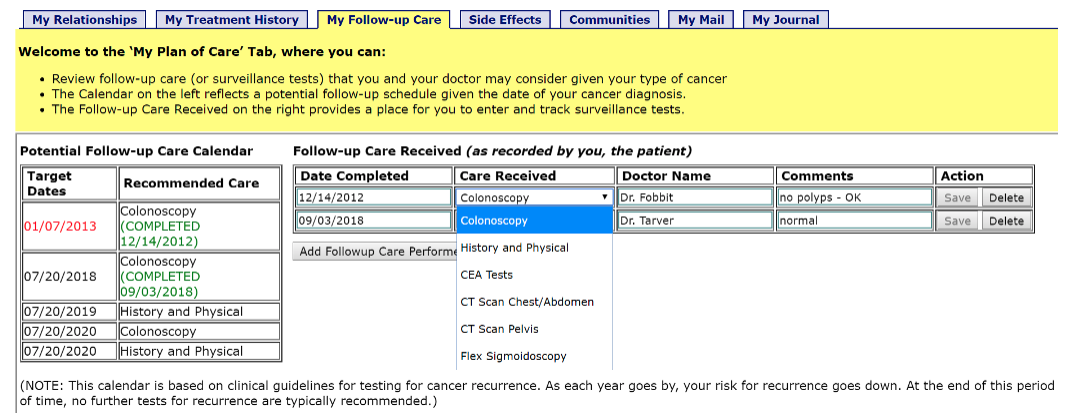
Screenshot of personal health record, “My Follow-up Care” Dashboard. CEA: carcinoembryonic antigen; CT: computed tomography.

Surveillance guidelines included information about guideline-concordant surveillance care, including bowel surveillance (colonoscopy), CEA tests, and CT scans [2], with reminders for individual surveillance tests based on individuals’ needs. Recommendations for surveillance care were adapted from the guidelines of professional organizations [2]. Information about CRC surveillance guidelines were automatically tailored to the CRC survivor’s disease stage (eg, CEA testing was not to be recommended for patients with stage I CRC). In the CRC-PHR, 2 tables related to surveillance care were delivered to the patient (Figure 1). First, a table was generated, which indicated what surveillance tests were appropriate for the patient and by what dates the tests should be performed. This informational table was designed to increase patient knowledge about surveillance and its benefits, as well as to prompt CRC survivors to seek the receipt of surveillance tests [35]. Second, a table was created wherein patients could self-enter information about the surveillance test received (date completed, type of test, and a brief description of results). This interactive table was designed both to enable the tracking of completed tests and to promote patient self-efficacy; interactivity is widely believed to enhance user involvement, commitment, and learning [36].

A 30-minute training session was developed to introduce patients to the content and functionality of the CRCS-PHR and was conducted in person by the research assistant at the time of recruitment. In addition, virtual training tools were embedded in the CRCS-PHR, including a 5-minute narrated training video and a detailed help section describing the purpose of all links, data-entry forms, and features of the tool.

### Study Design

A pre- and posttest intervention trial was conducted to test the feasibility and to determine the ability of the targeted PHR intervention to increase patient knowledge, self-efficacy, beliefs, and receipt of surveillance tests among CRC survivors. Participants were recruited between March and October 2012, with the postintervention follow-up survey occurring 6 months after the baseline survey.

### Sample and Recruitment

Patients with CRC were eligible to participate if they had received curative-intent therapy and had been diagnosed with American Joint Committee on Cancer Stage I-III adenocarcinoma at least 9 months (but no more than 24 months) earlier. Participants were excluded from the study if they had metastatic disease. We approached CRC survivors for recruitment at an academic medical center and the Veterans Affairs (VA) hospital in Indianapolis. At the academic medical center, patients were seen in surgery clinics led by CRC surgeons.

### Data Collection and Measures

Data were collected via patient self-report. Self-reported data are a valid, widely accepted source about clinical service use due to cost and time efficiency, particularly for early-phase studies such as this feasibility study, as well as large-scale epidemiologic studies [37-40]. Presurvey measurements were collected by a research assistant at baseline immediately after the patient was provided with access to the CRCS-PHR. Postsurvey measurement was then collected 6 months after initial enrollment over the telephone, with a written survey being mailed beforehand to patients in order to facilitate answering the questions. In addition to the measures discussed below, participants were asked how they used the PHR and what features they found to be most and least useful after the intervention.

### Measurements

#### Patient and Clinical Characteristics

Patient sociodemographic characteristics were collected during the baseline survey. Clinical characteristics regarding anatomic cancer site (colon or rectum), stage, and treatment (surgery, radiation treatment, and chemotherapy) were collected via medical record audit at the time of enrollment.

#### Patient-Centered Behavior Outcomes

Patient-centered behavior outcomes were all collected during the pre- and postintervention surveys. These outcomes included self-efficacy, perceived benefits of surveillance testing, barriers to adherence of surveillance testing, and knowledge of CRC surveillance testing. For self-efficacy, the General Self-Efficacy (GSE) scale was used, which consists of 10 items using a 4-point Likert scale (Multimedia Appendix 1) [41]. The GSE scale measures general self-efficacy and has been translated into more than 30 languages. The GSE scale had a Cronbach alpha of .86 in the current sample. For perceived benefits of CRC surveillance testing, a 5-item Likert scale was used, with the last 3 items each including a question dedicated to each of the 3 surveillance tests (colonoscopy, CEA test, and CT scan). For barriers to adherence to surveillance testing, a 3-item Likert scale was used. The items regarding perceived benefits and barriers were drawn from domains originally identified as being related to CRC screening by Rawl et al [42,43] among first-degree relatives of patients with CRC.

#### Surveillance Receipt

Adherence to surveillance testing was captured via patient report during both the pre- and postintervention surveys. Patients were asked yes or no if they had undergone each of the following tests since having CRC surgery: colonoscopy, CEA test, and CT scan.

#### Patient Knowledge

Patient knowledge regarding follow-up surveillance tests and visits was assessed by asking participants how often they believe each surveillance test (colonoscopy, CEA test, CT scan, and physical examination) should be performed. Participants were given various time frame categories to choose from. The guideline-concordant test frequency is as follows: physical exam, 3-6 months; CEA test, 3-4 months; colonoscopy, 2-3 years; and CT scan, annually [2]. For each participant, the number of items answered correctly was summed to generate the knowledge score (0-4). Patient knowledge was assessed at both baseline and after the intervention.

#### Statistical Analysis

Frequencies and measures of central tendency were calculated for patient sociodemographic variables. Descriptive statistics were calculated for sociodemographics, patients’ beliefs about surveillance tests categories (knowledge, self-efficacy, barriers, and benefits), and receipt of surveillance tests (colonoscopy, CEA test, and CT scan). Paired *t* test (2-tailed) was used to examine the differences in patient-centered behavior outcomes pre- and postintervention delivery. To examine the differences in surveillance receipt before and after the intervention, McNemar test was used. Patients who did not complete the follow-up survey were excluded from the primary analysis. The excluded participants were compared to those who completed both surveys in terms of sociodemographic and clinical characteristics; no differences were found. Data were analyzed using STATA 16.1 (StataCorp).

### Ethical Considerations

Approval for this study was obtained by the Indiana University Institutional Review Board (1201007805), as well as the Indianapolis VA Research & Development committee. The procedures used in this study adhere to the guidelines of the World Medical Association Declaration of Helsinki. Prior to enrollment in the study, the purpose of the study and each participant’s role were explained. Written consent was obtained from everyone who participated.

## Results

### Patient Characteristics

A total of 28 patients with CRC completed the baseline survey, with 22 patients completing the follow-up survey at 6 months after the intervention. The majority of the sample was recruited at Indiana University Health (25/28, 89%), with 3 patients being recruited through the Indianapolis VA Medical Center. Table 1 describes the patient sociodemographics. The average age of the sample was 58 (SD 9.9) years. Two-thirds (18/28, 64%) of the patients had rectal cancer vs colon cancer (9/28, 33%). The majority of the patients were male (16/28, 57%), married (20/28, 71%), and were employed full-time (15/28, 54%); they also had an annual household income of >US $60,000 (16/28, 57%). Slightly less than half of patients (13/28, 46%) had a college education or greater.

**Table 1:** Demographic characteristics (N=28).

Characteristics		Values
Age (years), mean (SD)		58 (10)
**Sex, n (%)**		
	Male	16 (57)
	Female	12 (43)
**Cancer type, n (%)**		
	Colon	9 (32)
	Rectal	18 (64)
	Unknown	1 (4)
**Education, n (%)**		
	High school	8 (29)
	Some college or trade school	7 (25)
	Associate or bachelor’s degree	7 (25)
	Some or complete graduate school	6 (21)
**Current marital status, n (%)**		
	Married (or long-term commitment)	20 (71)
	Not Married	8 (29)
**Employment status, n (%)**		
	Full-time	15 (54)
	Part-time	1 (4)
	Unemployed	3 (11)
	Retired	7 (25)
	Unable to work	2 (7)
**Income (US$), n (%)**		
	<30,000	5 (18)
	30,001-59,999	7 (25)
	>60,000	16 (57)

### Patient-Centered Behavior Outcomes

Descriptive statistics and paired *t* test results for the 4 categories of behavior outcomes, including self-efficacy, perceived benefit, perceived barriers, and patient knowledge, both before and after the intervention are reported in Table 2, while Tables 3-5 provide a more in-depth view of how patients answered the baseline questions for the 3 categories of knowledge, barriers, and benefits. For knowledge, patients were asked about the correct intervals of recommended follow-up times for various surveillance tests (physical exam, CEA test, colonoscopy, and CT scan). Out of the 4 knowledge questions, patients answered on average just under 2 of the 4 correctly, with no change in knowledge between the two surveys (*P*=.69). Self-efficacy (range 10-40) saw little change between pre- (32.2) and postintervention surveys (31.8; *P=.*66). Patients rated barriers (range 3-15) at both intervals fairly low, with 4.7 before the intervention and 4.9 at after the intervention *P*=.81). Benefits (range 5-25) is the only beliefs category in which we saw a significant change, and this was only benefits for CEA test, as benefits for all tests were rated fairly high. For colonoscopy, patients rated benefits before the intervention at 22.6, with no change after the intervention at 22.8 (*P*=.75). CEA test was rated as 20.9 before the intervention, with 22.0 after the intervention (*P*=.04). CT scan saw little change with preintervention rating at 21.6 and postintervention rating at 22.1 (*P*=.45).

**Table 2:** Patients’ beliefs about surveillance tests (n=22).

Patients’ beliefs			Before intervention, mean (SD)		After intervention, mean (SD)		*P* value	
Knowledge (range: 0-4)			1.7 (1.1)		1.7 (1.1)		.69	
Self-efficacy (range: 10-40)			32.2 (3.6)		31.8 (3.4)		.66	
Barriers (range: 3-15)			4.7 (2.2)		4.9 (1.9)		.81	
**Benefits (range: 5-25)**								
	Colonoscopy	22.6 (2.6)		22.8 (2.3)		.75		
	CEA^a^ test	20.9 (3.3)		22.0 (2.9)		*.04^c^*		
	CT^b^ scan	21.6 (2.7)		22.1 (2.6)		.45		

^a^CEA: carcinoembryonic antigen.

^b^CT: computed tomography.

^c^Italicized *P* values indicate significant value at the .05 level.

**Table 3:** Response frequency for benefits and barriers of surveillance at baseline.

Questions and responses				Strongly disagree		Disagree		Neither		Agree		Strongly agree
**Benefits**												
	Finding the recurrence of CRC^a^ early will save your life.			0 (0)		0 (0)		0 (0)		7 (25)		21 (75)
	The treatment for the recurrence of CRC may not be as bad if the cancer is found early.			0 (0)		2 (7)		0 (0)		11 (39)		15 (54)
	**The following tests will help find the recurrence of CRC early:**											
		Colonoscopy	0 (0)		0 (0)		0 (0)		6 (21)		22 (79)	
		CEA^b^ test	0 (0)		1 (4)		6 (21)		11 (39)		10 (36)	
		CT^c^ scan	0 (0)		0 (0)		2 (7)		14 (50)		12 (43)	
	**The following tests will decrease your chances of dying from the recurrence of CRC:**											
		Colonoscopy	1 (4)		1 (4)		1 (4)		8 (28)		17 (60)	
		CEA test	1 (4)		0 (0)		8 (29)		10 (36)		9 (32)	
		CT scan	1 (4)		0 (0)		2 (7)		14 (50)		11 (39)	
	**The following tests will help you not worry as much about the recurrence of CRC:**											
		Colonoscopy	2 (7)		1 (4)		0 (0)		7 (25)		18 (64)	
		CEA test	2 (7)		0 (0)		6 (21)		11 (39)		9 (32)	
		CT scan	2 (7)		0 (0)		1 (4)		13 (46)		12 (43)	
**Barriers**												
	You feel anxious about having follow-up tests because you don't really understand what will be done.			16 (57)		9 (32)		0 (0)		3 (11)		0 (0)
	The cost would keep you from having follow-up tests.			12 (43)		14 (50)		0 (0)		1 (4)		1 (4)
	Transportation problems would keep you from having follow-up tests.			15 (54)		10 (36)		1 (4)		2 (7)		0 (0)

^a^CRC: colorectal cancer.

^b^CEA: carcinoembryonic antigen.

^c^CT: computed tomography.

**Table 4:** Response frequency for knowledge about surveillance at baseline^a^.

Questions and responses				3-4 Months		6 Months		Yearly		Never		Don’t know
**Knowledge**												
	**How often do you believe the following cancer surveillance tests should be performed for a colon cancer survivor similar to yourself?**											
		Physical examination	*14 (50)*		*7 (25)*		0 (0)		6 (21)		1 (4)	
		CEA^b^ test	*14 (50)*		7 (25)		0 (0)		1 (4)		6 (21)	

^a^Italicized responses are the correct answers to the frequency for each surveillance test.

^b^CEA: carcinoembryonic antigen.

**Table 5:** Response frequency for knowledge about surveillance at baseline (continued)^a^.

Questions and responses			Yearly	2-3 Years	4-5 Years	Never	Don’t know
**Knowledge**							
	**How often do you believe the following cancer surveillance tests should be performed for a colon cancer survivor similar to yourself?**						
		Colonoscopy	20 (71)	*5 (18)*	2 (7)	0 (0)	1 (4)
		CT^b^ scan	*16 (57)*	3 (11)	2 (7)	0 (0)	7 (25)

^a^Italicized responses are the correct answers to the frequency for each surveillance test.

^b^CT: computed tomography.

### Receipt of Surveillance Testing

Table 6 reports the prevalence and comparison (paired *t* test) for each of the 3 primary surveillance tests at each time point. Since having surgery, only 52% had a colonoscopy since their CRC surgery, while that number increased to 86% (18/21) after the intervention (*P*=.005). Similarly, 67% (14/21) had a CEA test from the time of their surgery prior to the preintervention survey, while the proportion increased to 95% (20/21) after the intervention (*P=.*01). CT scan was the only surveillance test in which we did not see a significant uptick, with 67% (14/21) reporting having had a CT scan at the beginning of the study and 86% (18/21) having had one after the intervention (*P*=.10).

**Table 6:** Receipt of surveillance tests (n=21).

Tests	Preintervention, n (%)	Postintervention, n (%)	*P* value
Colonoscopy	11 (52)	18 (86)	*.005^c^*
CEA^b^ test	14 (67)	20 (95)	*.01*
CT^c^ scan	14 (67)	18 (86)	.10

^a^Italicized *P* values indicate significant value at the .05 level.

^b^CEA: carcinoembryonic antigen.

^c^CT: computed tomography.

## Discussion

### Overview

The purpose of our study was to test the feasibility of a stand-alone PHR for CRC survivors’ post resection and examine its impact upon receipt of recommended surveillance testing and behavior outcomes. CRC survivors are at an increased risk of recurrence, especially within the first 2-3 years after treatment. Interventions targeted toward increasing surveillance rates for CRC survivors would help to detect signs of recurrence early in its progression, and thus potentially decrease morbidity and mortality.

### Principal Findings

For our primary outcome, we found an overall significant impact on receipt of CRC surveillance tests. From baseline to the 6-month postintervention follow-up, we saw a significant impact for both colonoscopy (*P*=.005) and CEA testing (*P*=.01). There was no significant increase in CT scans between before and after the intervention, although we did observe an increase from 67% (14/21) at baseline to 86% (18/21) of the sample receiving a CT scan at 6 months. The effect of CRC surveillance tests is commonly clinically approached as a bundle of care, that is, recommending the combination of colonoscopy, CEA testing, and imaging [44]. At baseline, only 42% (12/28) of participants reported having received all 3 tests, with 29% (8/28) not having received any. Whereas, at the 6-month follow-up, 77% (16/21) had received all 3 surveillance tests, and all patients had received at least one. Both CEA testing and CT scans have been associated with increased rates of surgical treatment of recurrence, suggesting that increases in either type of surveillance testing may be associated with more salvage surgery with curative intent [45].

### Comparison With Prior Work

Existing frameworks provide some guidance about what behavioral mechanisms may explain these main effects. The Health Belief Model [46] posits that self-efficacy, perceived barriers, and perceived benefits (ie, belief about the effectiveness of surveillance in reducing risk) mediate changes in the health behavior of individuals. Further, other investigators have postulated that patient-centered portals, with many features in common with personal health records, will have a positive effect upon patient self-efficacy [47-49]. In addition, Lo et al [50] found screening knowledge, perceived barriers to care, and social norms to be significant mediators of sociodemographic differences in the uptake of CRC screening. The CRCS-PHR may act through similar mechanisms. Our patient-centered technology had design features intended to increase screening knowledge and the perceived benefit of surveillance, including both clinical reminders to the patients about the next surveillance test due and web-based educational materials to explain the nature and purpose of each test. Participants found the summary and schedules of their cancer treatment and follow-up appointments to be the most useful in the PHR, along with side effects of treatment and community resources [31,47].

Due to the conceptual and empiric importance of patient knowledge, perceived benefits and barriers, as well as self-efficacy to the uptake of CRC screening [51], we explored the effect of the CRCS-PHR upon these secondary outcomes. Many patients did not know the answer to individual knowledge questions (Table 3); the proportion varied by test, from 25% (7/28) to 75% (21/28) for physical exam to colonoscopy, respectively. These findings suggest that patient-centered technologies have the potential to increase patient knowledge but can be further tailored to tests about which patient have the least awareness (eg, colonoscopy or CT scans). Nonetheless, patient knowledge is commonly not associated with changes in patient screening behavior [52]; our observations that surveillance test use increased, whereas knowledge about the tests often did not, reinforced this weak association.

Overall, patients largely agreed about the benefits of CRC surveillance, with the proportion who reported individual tests (colonoscopy, CEA test, or CT scan) as beneficial ranging from 75% to 100% (find recurrence early); from 68% to 88% (decrease chance of dying from recurrence); and from 71% to 89% (help you not to worry). With patient beliefs, the only domain that significantly increased was the perceived benefits of CEA testing, as most were quite high at baseline. These relatively high proportions are similar to the perceived benefit of CRC screening tests among a general population not already diagnosed with cancer [43]. High perceived benefit limited the potential for improvement in these perceptions among the CRC survivors enrolled in our trial. Conversely, low perceived barriers to care at baseline likely limited the potential for improvement in these domains. Moreover, the potential barriers of cost and transportation are challenging to address [53-58], and our CRCS-PHR implementation needs to be accompanied by changes in the health care systems, and policy needs to be adequately addressed.

We found no significant differences in patient self-efficacy. Systematic reviews conducted by Han et al [47] and Lancaster et al [48] found that eHealth tools such as provider-patient communication functionalities, case management, and other forms of clinical support may increase self-efficacy and self-management. Empiric findings from previous studies have been mixed. Secure messaging had a positive impact upon medication self-efficacy among patients with diabetes [59,60], but other studies have shown no association [60]. These mixed findings suggest that the influence of patient portals upon self-efficacy may vary depending upon both the functions used and the populations targeted.

Future studies in the field of cancer control should assess new populations of patients with cancer, including prostate, ovarian, and skin cancers, as they are underrepresented in this area of the literature and patient-centered technology [31]. However, with differences across sites of cancer, and more specifically, across the cancer continuum, patient-centered technology interventions will need to be targeted toward specific sites and continuum levels as efficacy and effectiveness may vary [31]. This may be due in part to the complexity of cancer care across the care trajectory versus other chronic disease management, as well as the need for tailored functionalities by cancer type. Treatment plans and surveillance testing will likely differ for each cancer type in terms of the tests and intervals recommended. Future studies should also consider what types of recruitment strategies may be optimal in this type of research, such as recruitment through clinicians versus registry-based outreach. We employed a recruitment strategy involving clinician engagement to recruit individuals, as we felt this approach would better identify eligible patients and decrease attrition over time due to ongoing engagement with their clinicians. Testing this type of engagement was important to assess initial intervention feasibility. However, there are advantages to registry-based recruitment, which is more likely to lead to increased access to a larger number of potential participants. Researchers should weigh the advantages and disadvantages of different recruitment strategies in relation to the specific needs of their studies.

### Limitations

While this work provides strong insights and evidence to inform the development of the CRCS-PHR, our work is not without its limitations. First, without a control group not exposed to the CRCS-PHR, we are limited in our ability to make inferences of the intervention effect. Additionally, the pre- and posttest design has the potential for a temporal effect on the patient beliefs and surveillance receipt over time, which is important to note. With feasibility established, future studies should use a randomized controlled trial design, which will account for the potential of a temporal effect and increase the strength of our causal inferences with the introduction of a control group. The relatively small sample size of our feasibility study also limits our ability to test mediation pathways. Our sample was primarily White, younger on average compared with the national CRC population (58 years versus 66 years old) [1], and more highly educated than the population of Indiana and that of the United States (13/28, 46% of the sample having college degree). The young age and higher education of our sample is not unusual among early adopters of new patient technologies and cancer survivors recruited at academic medical centers [60-62]; however, the results are not directly generalizable to other populations, including patients treated in community-based oncology clinics. Future work needs to continue to focus upon how best to engage CRC survivors who tend to be older adults in use of these new technologies. While our sample had a majority of rectal patients, nearly an inverse of the US CRC survivor population (70% of national CRC survivors being colon), we believe it is important to understand the use of this technology among both rectal and colon cancer survivors, and do not have a reason to believe its use would differ between these closely related cancer types. The higher proportion of rectal to colon patients in the sample was due in part to the local expertise in rectal cancer surgery at one of the academic sites.

### Conclusions

Patient-centered technologies such as the CRCS-PHR represent an important potential approach to improving the receipt of guideline-concordant care such as surveillance tests among cancer survivors. In assessing these rapidly emerging technologies, we encourage investigators and evaluators to continue measuring behavioral constructs that might serve as plausible mechanisms to explain observed effects. With this approach, we can grow to understand not only if new technologies improve the quality of care but how this improvement takes place. Future research in this area should also assess the effect of personal health records with quasi-experimental and randomized controlled study designs when possible. Finally, survivors of different types of cancers should be enrolled in future research, given that the clinical and supportive care needs of patients may vary widely among different populations.

## Appendix

Multimedia Appendix 1 General self-efficacy measure.

## Abbreviations

CEA: carcinoembryonic antigen
CRC: colorectal cancer
CRCS: colorectal cancer survivors
CRCS-PHR: Colorectal Cancer Survivor Personal Health Record
CT: computed tomography
GSE: General Self-Efficacy
PHR: personal health record
VA: Veterans Affairs
