# Supplementary material for: Impact of a Personal Health Record Intervention Upon Surveillance Among Colorectal Cancer Survivors: Feasibility Study
Source: JMIR Cancer. 2022 Aug 11;8(3):e34851. doi: 10.2196/34851 (PMC9412760; doi:10.2196/34851)
Supplement: Multimedia Appendix 1 [file cancer_v8i3e34851_app1.docx]

**Multimedia Appendix 1: General self-efficacy measure**

For each question, answer: “Not at all true”; “Hardly true”; “Moderately true”; and “Exactly true.”

1. I can always manage to solve difficult problems if I try hard enough.
2. If someone opposes me, I can find the means and ways to get what I want.
3. It is easy for me to stick to my aims and accomplish my goals.
4. I am confident that I could deal efficiently with unexpected events
5. Thanks to my resourcefulness, I know how to handle unforeseen situations.
6. I can solve most problems if I invest the necessary effort.
7. I can remain calm when facing difficulties because I can rely on my coping abilities.
8. When I am confronted with a problem, I can usually find several solutions.
9. If I am in trouble, I can usually think of a solution.
10. I can usually handle whatever comes my way.
